# Trapped in the web of water: Groundwater‐fed springs are island‐like ecosystems for the meiofauna

**DOI:** 10.1002/ece3.2535

**Published:** 2016-10-25

**Authors:** Simone Fattorini, Paulo A. V. Borges, Barbara Fiasca, Diana M. P. Galassi

**Affiliations:** ^1^ Department of Life, Health and Environmental SciencesUniversity of L'AquilaL'AquilaItaly; ^2^ Departamento de Ciências e Engenharia do AmbienteCE3C—Centre for Ecology, Evolution and Environmental Changes/Azorean Biodiversity GroupUniversidade dos AçoresAngra do HeroísmoAçoresPortugal

**Keywords:** beta diversity, copepods, equilibrium theory, groundwater, island biogeography, nestedness

## Abstract

We investigated whether the equilibrium theory of island biogeography (ETIB) can be applied to the meiofauna of groundwater‐fed springs. We tested whether copepod species richness was related with spring area, discharge, and elevation. Additionally, five hypotheses are tested based on species distribution patterns, dispersal ability, and life‐history characteristics of several guilds (stygobiotic, nonstygobiotic, cold stenotherm, and noncold stenotherm species). Thirty springs in the central Apennines (Italy) were considered. A multimodel selection procedure was applied to select best‐fit models using both ordinary least‐squares regressions and autoregressive models. Mantel tests were used to investigate the impact of spatial autocorrelation in determining interspring similarity (ßsor), pure turnover (ßsim), intersite nestedness (ßnest = ßsor − ßsim), and matrix nestedness (measured using NODF and other metrics). Explicit consideration of spatial correlations reduced the importance of predictors of overall species richness, noncold stenotherm species (both negatively affected by elevation), cold stenotherm species, and nonstygobiotic species, but increased the importance of area for the stygobiotic species. We detected nested patterns in all cases, except for the stygobites. Interspring distances were positively correlated with ßsor and ßnest (but not with ßsim) for the entire data set and for nonstygobiotic, cold stenotherm, and noncold stenotherm species. In the case of stygobites, interspring geographical distances were marginally correlated with ßsor and no correlation was found for ßsim and ßnest. We found support for ETIB predictions about species richness, which was positively influenced by area and negatively by elevation (which expresses the size of source of immigrants). Low turnover and high nestedness are consistent with an equilibrium scenario mainly regulated by immigration and extinction. Stygobites, which include many distributional and evolutionary relicts, have a low capability to disperse through the aquifers and tend to be mainly confined to the springs where they drifted out and were trapped by springbed sediments.

## 1 Introduction

The equilibrium theory of island biogeography (ETIB) proposed by MacArthur and Wilson (1963, 1967) to explain variation in species number on islands turned into one of the most productive research programs in ecology (Lomolino, 2000; Lomolino, Riddle, Whittaker, & Brown, 2010; Warren et al., 2015). Providing a simple mechanistic model based on extinction and colonization rates, the ETIB obtained an immense success even far beyond its original scope, being applied to virtually any kind of isolates (Walter, 2004). The widespread implications of the ETIB were already clear to MacArthur and Wilson themselves, who stated that the principles of island biogeography could be applied to any kind of isolated habitats, such “streams, caves, gallery forest, tide pools, taiga as it breaks up in tundra, and tundra as it breaks up in taiga” (MacArthur & Wilson, 1967: 3–4).

Despite the ETIB has reached the status of a scientific paradigm, few studies have explicitly tested its foundations (Whittaker & Fernández‐Palacios, 2007), because of obvious difficulties in measuring colonization and extinction rates. Thus, most research that evoked the ETIB has been based on the assumed relationships between geographical parameters and extinction/colonization rates. For example, it is usually hypothesized that immigration rates should decrease with increasing distance of islands from the mainland and/or other islands and increase with island size (the so‐called target effect, because larger islands are larger targets) (Lomolino et al., 2010; Warren et al., 2015; Whittaker & Fernández‐Palacios, 2007). At the same time, it is assumed that extinction rates should decrease with increasing island size, because larger islands host larger, and hence more stable populations (Lomolino et al., 2010; Warren et al., 2015; Whittaker & Fernández‐Palacios, 2007), and that extinction rates should also decrease with island proximity due to rescue effects (Brown & Kodric‐Brown, 1977).

Although springs have been repeatedly suggested as isolates (e.g., Cantonati, Füreder, Gerecke, Jüttner, & Cox, 2012; Davis, Pavlova, Thompson, & Sunnucks, 2013; Glazier, 2009; Spitale & Petraglia, 2009; Stutz, Shiozawa, & Evans, 2010; Teittinen & Soininen, 2015; Werum, 2001), no study has so far explicitly tested the ETIB for groundwater organisms inhabiting springs. In fact, the only study that attempted to apply ETIB principles to springs is a recent analysis of the diatoms of boreal springs, where species richness was not influenced by area and isolation (Teittinen & Soininen, 2015). This study, however, dealt with a group of unicellular organisms with high dispersal capabilities (but see Keleher & Rader, 2008 for reduced dispersal in metaphyton communities of desert springs).

Springs are currently classified as groundwater‐dependent ecosystems (Eamus & Froend, 2006), yet they are usually approached under an epigean perspective, and subsurface spring habitats have been almost completely neglected (Fiasca et al., 2014; Stoch et al., 2016). This appears surprising, because groundwater‐dependent ecosystems are becoming key research topics in freshwater biology and ecology (Howard & Merrifield, 2010). Springs may be considered as natural windows to the subterranean world, thus representing unique environments to investigate the ecology and biogeography of groundwater organisms which reach the springs from the aquifers where they spend the whole life cycle (Galassi et al., 2014). However, most of the researches on spring ecology have dealt with plants and animals living in the spring head above the bottom substratum, with only a few studies investigating the spring subsurface meiofauna (Fiasca et al., 2014). Moreover, these studies dealt mainly with the influence of physicochemical parameters on spring community structure (Fiasca et al., 2014), whereas no study has been addressed to the biogeography of the groundwater meiofauna inhabiting the springs. There is increasing evidence that springs are geographical isolates of conservation concern for the high levels of cryptic diversity of both surface‐water and groundwater macroinvertebrates (Murphy et al., 2015), even if the explanatory factors shaping differentiation of cryptic species and micro‐endemicity are still matter of debate (Juan et al., 2010; Rader et al., 2012). In this study, we aimed at testing—for the first time—the principles of ETIB to groundwater invertebrate meiofauna using explicitly stated hypotheses.

**Figure 1:**
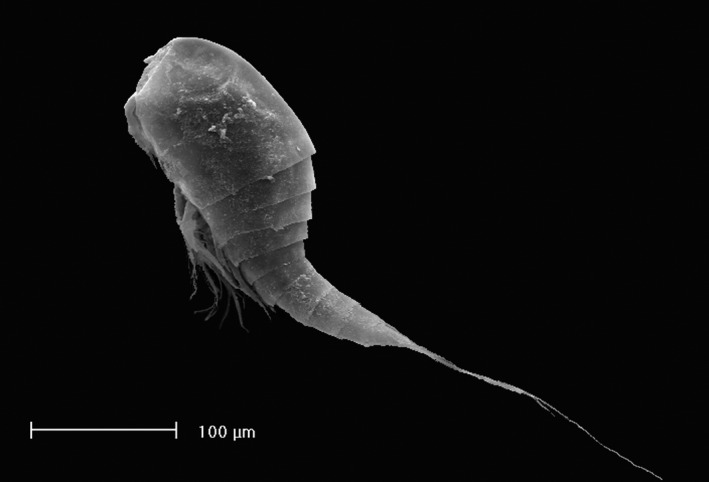
The stygobiotic copepod crustacean *Pseudectinosoma reductum* Galassi & De Laurentiis, 1997. It was discovered in the Presciano spring system (Gran Sasso aquifer) and after recorded also from the Cavuto spring (Montagna Grande aquifer). This species is a “living fossil” of ancient marine origin, with a relict distribution and a very isolated phylogenetic position

We considered two levels of diversity: species richness recorded in each spring and β‐diversity (spatial turnover) of copepods (Crustacea, Copepoda, Figure 1). According to the principles of ETIB, we postulate that species richness should increase with spring area and decrease with isolation. Also, we expect a distance decay effect, that is, that interspring similarity in species composition should decrease with increasing interspring distances, which should lead to a correlation between β‐diversity and geographical distances (Nekola & White, 1999).

Dynamic models, such as the ETIB, suppose that distribution patterns on islands are mainly influenced by present conditions, such as island distance to the mainland, interisland distances, and habitat heterogeneity, more than by historical factors. However, some species may display distributional patterns that are the result of past events, and relict models postulate that species distribution on islands was determined by past interisland and/or island–mainland connections followed by vicariance events, instead of present conditions, such as island distance to the mainland (see Whittaker & Fernández‐Palacios, 2007).

The relative influence of current and historical factors varies according to the island systems and the organisms. In general, species with low dispersal capabilities show distributional patterns more strongly constrained by regional historical factors (such as geological events, paleogeographical setting, and paleoclimatical changes), whereas present local and regional ecological factors may be more important for taxa with higher dispersal abilities.

According to the ETIB, the species–area relationship is a consequence of the fact that extinction rates decrease and immigration rates increase with island area. By contrast, according to relict models, a positive species–area relationship results from area‐dependent extinction or relaxation processes.

Stygobites (i.e., organisms that complete their whole life cycle in groundwater) have lower dispersal capabilities in comparison with nonstygobiotic species (Galassi, Huys, & Reid, 2009; Stoch & Galassi, 2010; Trontelj et al., 2009), and their distributional patterns are recognized to result mainly from past geological events (but see Eme et al., 2015 for the role of spatial heterogeneity and productive energy). Thus, stygobites should form “relict” assemblages (Holsinger, 1988; Humphreys, 2000; Galassi, Dole‐Olivier, & De Laurentiis, 1999; Galassi et al., 2009), whereas nonstygobiotic species are expected to be more influenced by current ecological factors that can affect their dispersal.

On the basis of these considerations, we formulated the following predictions:

1. Species richness should correlate positively with spring area for both nonstygobites and stygobites. However, we hypothesize that, if stygobiotic species form relict assemblages, their richness should not be significantly influenced by other variables, such as discharge and elevation, which may be important for species which conform to the ETIB.
2. If stygobites form relict populations, they should have idiosyncratic distributions that are not strongly influenced by current geography; thus, we expect that their species richness values should not be strongly autocorrelated. By contrast, if nonstygobites follow an equilibrium model, with species exchanges facilitated by interspring proximity, we expect a strong spatial autocorrelation in species richness values for these animals.
3. Because of their idiosyncratic distributions, stygobiotic species should present low nestedness. By contrast, if nonstygobites are subject to continuous processes of immigration/extinction, we expect significant nestedness.
4. Noncold stenotherm and nonstygobiotic species are known to have higher dispersal capabilities compared to stygobites, and hence, we hypothesize that they can move easily through the surface hydrogeological network at low altitude. So, we expect that noncold stenotherm and nonstygobiotic species richness are negatively related to spring altitude. Moreover, these species should be more widespread and show lower spatial turnover.
5. Cold stenotherm species need a stable thermal regime. As higher groundwater discharge guarantees cold and stable thermal regime, we expect that cold stenotherm species correlate positively with discharge.

## 2 Methods

The studied springs are located in the central Apennines (Abruzzo region). Climate is continental, with average annual precipitation ranging from 700 to 1,000 mm. Springs are distributed in three main hydrogeological units represented by the Gran Sasso Massif, the Montagna Grande, and the Laga Mountains. The Gran Sasso karstic aquifer consists of small aquifer subunits divided by main structural discontinuities. The Montagna Grande is a hydrogeological system defined by dolomite deposits and is disconnected from the surrounding ones. The Laga Mountains are characterized by an arenaceous flysch complex; its low permeability limits the percolation of rainfall waters and enables surface runoff. The subsurface hydrological network of the Laga Mountains has low discharge and feeds springs and swamps.

**Figure 2:**
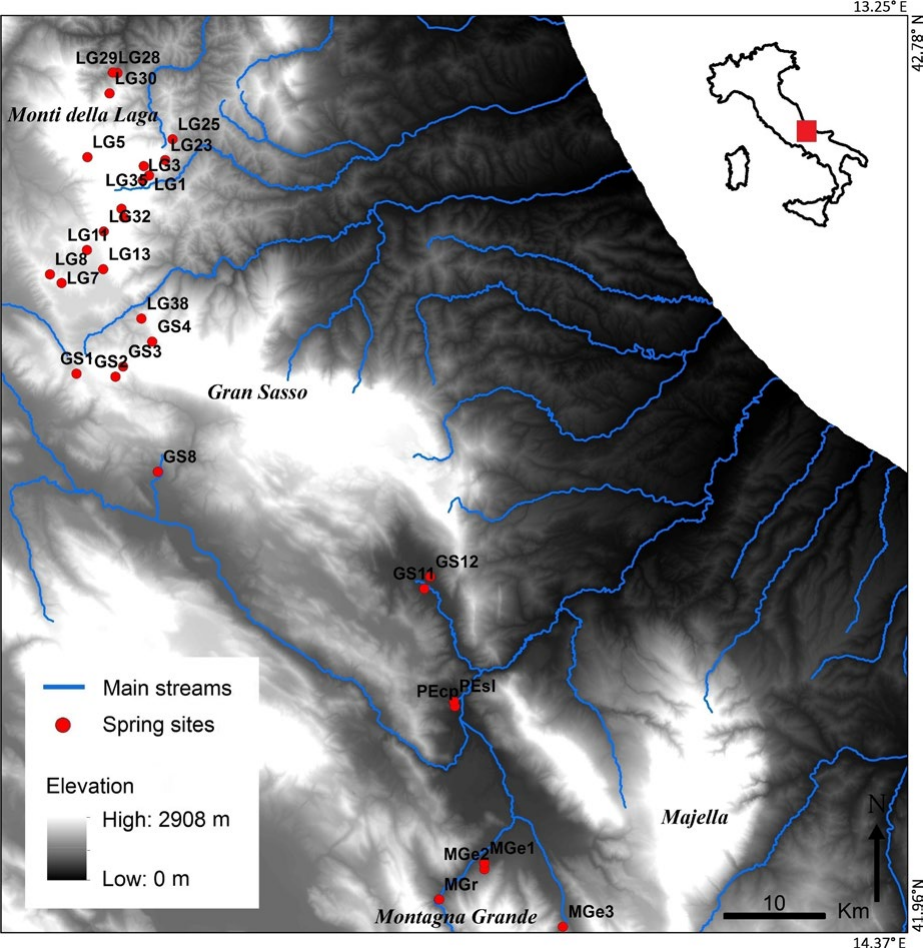
Map of the study area (Abruzzo mountains) with indication of sampled springs. Springs codes as in Table 1. Top right inset shows location of the study area in Italy

Thirty springs, fed exclusively by groundwater, were sampled across the three hydrogeological units analyzed (Figure 2). All the investigated springs are pristine, perennial, and located in undisturbed areas. A stratified sampling has been performed in each spring in order to cover most of the environmental heterogeneity. Sampling was carried out by means of drift nets placed at the major openings of the bedrock in the case of karstic springs; alternatively, when sediments were present, the interstitial habitat was sampled by pumping 10 L of interstitial water with the Bou–Rouch method (Bou & Rouch, 1967) and then filtered with a hand net (60‐μm‐mesh size). Among the invertebrates collected, the microcrustacean copepods were identified to species level and used as target group being by far the most abundant and species‐rich taxon among meiofaunal organisms. Values of species richness reported in Table 1 should be considered virtually complete (Galassi, Fiasca, & Del Tosto, 2011). Primary data are given in Appendix S1 in Supporting Information.

**Table 1:** Hydrological characteristics, geographical location, and meiofaunal species richness of 30 groundwater‐fed springs studied in Central Italy

Spring name	Code	*Lat*	*Lon*	*E*	*D*	*A*	*T*	*Sb*	*Cs*
Fonte Lorma	GS1	42.45861	13.35741	1,355	0.06	1.08	7	0	1
Capodacqua	GS11	42.27061	13.78279	340	3,000	300	18	5	6
Presciano	GS12	42.28164	13.78962	330	1,800	2,000	22	9	7
Sorgente San Franco	GS2	42.45641	13.40452	1,730	0.42	0	4	0	1
Fonte Rignitti	GS3	42.46588	13.41361	1,765	0.06	0	3	0	1
Sorgenti del Chiarino	GS4	42.48868	13.44843	1,250	6.5	2	5	1	1
Sorgenti del Vera	GS8	42.37192	13.45821	665	2.343	0.2	9	2	1
Sorgente dello Zingaro	LG1	42.63785	13.44079	1,261	0.1	0.05	5	0	1
Sorgente Scritta	LG11	42.56963	13.36697	1,480	0.03	0.5	4	0	1
Sorgente Mastrangelo	LG13	42.55282	13.38726	1,396	0.03	0.4	3	0	0
Sorgente Centofonti	LG16	42.60747	13.40823	1,873	0.05	9	6	0	2
Sorgente Mercurio Cesacastina	LG18	42.59973	13.41107	1,610	0.04	0.21	3	0	1
Sorgente Fioli	LG23	42.65185	13.46001	1,146	0.15	3	4	0	1
Sorgente Ceppo	LG25	42.67056	13.46868	1,250	0.04	20	4	0	0
Sorgente Fosso della Montagna	LG28	42.72969	13.39455	729	0.33	0.5	9	2	2
Fonte Iachina	LG29	42.72928	13.40024	880	0.3	0	4	0	1
Sorgente Storro Padula	LG3	42.63333	13.43333	1,278	0.38	0.35	3	0	1
Sorgente Maolaro	LG30	42.71075	13.39089	1,479	0.2	2	3	0	1
Sorgenti del Tronto	LG32	42.58679	13.38692	1,930	0.17	0.433	7	0	2
Sorgenti del Tordino	LG35	42.64614	13.43414	1,736	1.723	16.767	4	0	2
Sorgente Fontanino	LG38	42.50905	13.43496	1,260	0.01	0.8	4	0	0
Sorgente Campellino	LG5	42.65317	13.36545	1,375	0.08	0.2	3	1	2
Sorgente Campotosto 1	LG7	42.53982	13.33716	1,504	0.01	20	4	0	1
Sorgente Campotosto 2	LG8	42.54738	13.32265	1,498	0.07	20	6	0	1
Bugnara	MGe1	42.02499	13.85999	618	10	0.43	9	3	2
Prezza	MGe2	42.01944	13.86000	618	10	0.46	6	1	1
Gizio	MGe3	41.96860	13.95527	620	450	60	10	8	2
Cavuto	MGr	41.99166	13.80583	641.75	224.528	26.003	18	5	4
Capo Pescara	PEcp	42.16536	13.82176	240	6,500	1,000	21	10	4
Santa Liberata	PEsl	42.17007	13.82037	260	1,000	60	23	7	5

*Lat*, latitude (decimal degrees); *Lon*, longitude (decimal degrees); *E*, elevation (m); *D*, discharge (Ls^−1^); *A*, area (m^2^); *T*, total number of species; *Sb*, number of stygobiotic species; *Cs*, number of cold stenotherm species. Codes refer to Figure 2.

### 2.1 Statistical analysis

**Figure 3:**
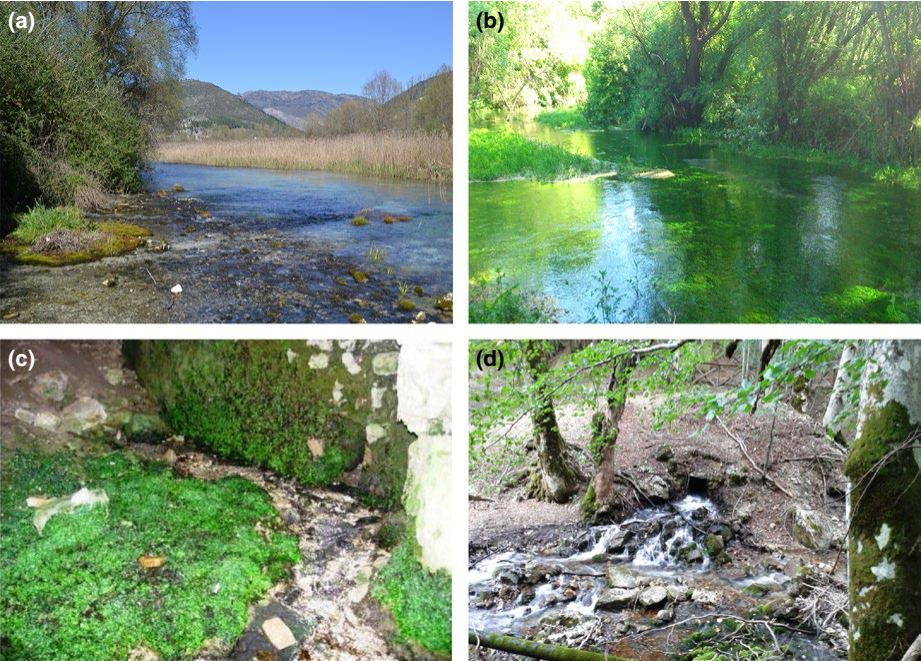
Examples of limnocrene (a, b) and rheocrene (c, d) springs. a: Presciano spring (GS12), b: Capo Pescara spring (PEcp), c: Cavuto spring (MGr), d: Chiarino spring (GS4). Codes as in Table 1 and Figure 2

Both the ETIB and relict models predict that species richness should increase with island area (species‐area relationship) and should decrease with isolation. We recorded spring area as the spring surface permanently covered by water. For larger, typically limnocrene springs (i.e., springs occurring where groundwater discharge from confined or unconfined aquifers emerges as one or more lentic pools; Springer & Stevens, 2008; Figure 3a,b), we measured water surface as the area of a polygon approximating spring surface using GIS facilities (QGIS 2.12 Development Team, 2012). For very small, typically rheocrene springs (i.e., springs where groundwater discharge emerges as flowing streams or rivulets to form a springbrook; Springer & Stevens, 2008; Figure 3c,d), we calculated water surface from linear measures (length × width) (see Teittinen & Soininen, 2015).

Measuring spring isolation is not a trivial procedure. Teittinen and Soininen (2015) used the proportion of terrestrial area in the catchment from the entire catchment area as a measure of spring isolation. This kind of measure does not seem appropriate for groundwater organisms. For true islands, isolation is usually measured as island distance to the mainland, which is assumed as the species source (Weigelt & Kreft, 2013). In the case of groundwater‐fed springs (i.e., springs fed exclusively by groundwater, in contrast to springs fed by rainfall, snowmelt, and glacier melt), the species source (i.e., the mainland) is the aquifer. Thus, a perfect analogous for springs should be the distance to the aquifer network, which is however impossible to measure, especially for the meiofauna. The animals that compose the meiofauna live not only in the small and large fractures filled by groundwater, but, in the case of karstic aquifers, also in the annex capacitive subsystem (Galassi et al., 2014), avoiding the large karstic conduits where water velocity is very high. Such patchiness in the distribution of the meiofauna, together with the intrinsic complexity of the aquifer network, makes it practically impossible to measure the groundwater flowpath from the aquifer to the spring. Thus, the subterranean pathways of water interconnections that can be used by the meiofauna remain unknown.

For these reasons, we opted for an indirect measure that might be viewed as a functional analogous of “distance to mainland.” We considered spring discharge as an inverse “functional” proxy of island–mainland distance. This is because a higher discharge, depending on the flow rate and the length of the flowpath from the recharge area, indicates a larger volume of water that is transferred from the aquifer to the spring, so that it should increase immigration rates of copepods. Mainland size is usually not considered in island biogeography (but see Weigelt & Kreft, 2013 for an isolation metric that accounts for the coastline shape of large landmasses by including only the area of the part that extends into a certain buffer). However, in the case of springs, it is important to consider that the proportion of the aquifer that represents the source pool of organisms is not the same even for springs that are fed by the same aquifer. Specifically, while high‐altitude springs receive water from a small portion of the aquifer (because of the proximity of the surface recharge area of the aquifer to the spring outlet), and thus may be likely colonized by individuals coming from a reduced source pool, basal springs are fed by a larger part of the aquifer and hence receive immigrants from a likely larger source pool.

In general, elevation is considered one the most important ecological factors shaping species richness patterns of copepod assemblages in springs, especially because stygobites are rare in high‐altitude mountain springs, whereas they represent an important fraction of the copepod species richness in springs located at lower altitudes (Stoch, 2007).

For these reasons, we introduced both discharge and elevation as possible factors influencing immigration rates, and hence species richness. We used few environmental predictors in our models even if other variables, such as spring temperature, shading, conductivity, pH, concentration of chemical parameters, or springbed sediment texture, might be important in determining species richness (Galassi et al., 2011; Reiss & Chifflard, 2015). However, we preferred to select only ecogeographical variables that paralleled those used in ETIB formulation, leaving the study of other correlates to future research.

Environmental variables were always log‐transformed to linearize their relationships with species richness values. The use of log‐transformed area values is also consistent with the Gleason exponential model of the species–area relationship: *S *= log*c* + *z* log *A*, where *S* is the number of species, *A* is the area, and *c* and *z* are fitted parameters (see Triantis, Guilhaumon, & Whittaker, 2012). For comparative purposes, we also applied the Arrhenius power model (log *S *= log *c *+ *z* log *A*; see Triantis et al., 2012), which however provided a slightly poorer fit.

Area was log (*x *+* *1)‐transformed because of the presence of zero values for springs so small that their surface was not possible to be measured with sufficient accuracy. We used a multimodel selection procedure to identify best‐fit models on the basis of the corrected Akaike information criterion (AICc). To investigate the impact of spatial autocorrelation on the relationships between environmental variables and species richness, we used both ordinary least‐squares regression (OLS) and spatial autoregressive (SR) models (Beale, Lennon, Yearsley, Brewer, & Elston, 2010; Fattorini & Ulrich, 2012; Liechstein, Simons, Shriner, & Franzreb, 2002) as implemented in SAM (Spatial Analyses in Macroecology) v. 4.0 software (Rangel, Diniz‐Filho, & Bini, 2010). Autocorrelation was evaluated using Moran's *I* index with 1,000 permutation to calculate *p*‐values. We conducted analyses for total species richness and for stygobiotic, nonstygobiotic, cold stenotherm, and noncold stenotherm species, separately.

To investigate correlation between interspring variation in species composition and interspring distances, we measured pairwise minimum surface distances between springs using QGIS (QGIS 2.12 Development Team, 2012). These distances express only the geographical proximity of the springs and not necessarily their degree of interconnection, which is influenced by the subterranean hydrology. In fact, springs that are geographically very far might be connected by subterranean networks, and geographically close springs may be hydrologically rather isolated. However, we used surface geographical distance as a measure of isolation under the assumption that interspring closeness should facilitate the exchange of individuals belonging to species with high dispersal power, although the reverse is not necessarily true (i.e., species with low dispersal capabilities might migrate from a spring to another if they are connected by the subterranean hydrology). We used the approach of Baselga, Jiménez‐Valverde, and Niccolini (2007) and Baselga (2010, 2012) for partitioning the overall ß‐diversity (ßsor) among springs into true species replacement or pure turnover (ßsim) and nestedness (ßnest) components. In this respect, nestedness quantified the part of compositional change caused by ordered species loss, whereas pure turnover was related to the exchange in faunal composition caused by the local trade‐off between species extinction and immigration. To assess whether variations in ßsor, ßsim, and ßnest were geographically structured, we correlated their values with interspring geographical distances using Mantel tests (Pearson correlation coefficient, with 10,000 random permutations to assess significance). Lack of significant correlations indicates that the considered species are unable to cross the surface landscape, whereas significant correlations may arise by both surface and underground dispersal.

To quantify the total degree of nestedness in each matrix, we used standard nestedness derived from the overlap and decreasing fill (NODF) metric (see Almeida‐Neto, Frensel, & Ulrich, 2012; Almeida‐Neto, Guimarães, Guimarães, Loyola, & Ulrich, 2008; Baselga, 2012), which is a normalized count of the degree of species overlap among the sequence of plots ordered according to decreasing species richness. For comparative purposes, we also calculated the following additional nestedness indices: matrix temperature, Brualdi and Sanderson discrepancy and spectral radius (Strona, Galli, Seveso, Montano, & Fattorini, 2014). We used a null model approach to assess whether observed patterns deviated significantly from random expectation (Gotelli & Ulrich, 2012) and compared observed scores with those obtained through randomization by referring to *Z*‐scores (which are calculated as [Nr − mean(Ns)]/stdev(Ns), where Nr is the nestedness of the matrix under study, and mean(Ns) and stdev(Ns) are, respectively, the average and standard deviation of the nestedness values of the null matrices). Although *Z*‐scores can be used to assess whether a matrix is significantly nested or not, they cannot provide information about the “magnitude” of nestedness (Strona, Stefani, Galli, & Fattorini, 2013). For this, we calculated also the “relative nestedness” (RN) proposed by Bascompte, Jordano, Melián, and Olesen (2003), which is computed as [Nr − mean(Ns)]/mean(Ns). Because species differ in regional abundances and therefore in colonization abilities (mass effects in the sense of Ulrich, Almeida‐Neto, & Gotelli, 2009 and Gotelli & Ulrich, 2012), we randomized the focal matrix using the fixed row‐equiprobable column model, which places species into the cells proportionally to observed total occurrences, as recommended by Gotelli (2000). Nestedness analyses were conducted using the NeD program (Strona et al., 2014) with 100 null matrices.

## 3 Results

We found significant spatial autocorrelations (Moran's *I* at the first distance class) for the complete species set (*I *=* *.439, *P*_*I*=0_ = .010), for nonstygobiotic (*I *=* *.302, *P*_*I*=0_ = .015), for cold stenotherm (*I *=* *.347, *P*_*I*=0_ = .014), and for noncold stenotherm (*I *=* *.455, *P*_*I*=0_ = .005) species. Stygobiotic species richness was not spatially autocorrelated (*I *=* *.142, *P*_*I*=0_ = .658), which supports our Prediction #2.

For the complete data set, the OLS best‐fit model included area and elevation as significant predictors (Table 2). Area, however, was not significant when the autoregressive model was applied (Table 2). By contrast, area was the only predictor in the best‐fit OLS model for stygobiotic species and was significant also in the autoregressive model (Table 2), which supports a relict model for stygobites (Prediction #1).

**Table 2:** Parameter values, standard errors and associated probability levels of ordinary least‐squares (OLS, a, b, c) and spatial autoregressive (SR, d, e, f) best‐fit models for total, stygobiotic and cold stenotherm species richness, and log‐transformed environmental variables

OLS models											
Total species richness				Stygobiotic species				Cold stenotherm species			
Variable	Coefficient	Standard error	*p*(t)	Variable	Coefficient	Standard error	*p*(t)	Variable	Coefficient	Standard error	*p*(t)
a				b				c			
Constant	52.085	7.177	<.001	Constant	1.394	0.589	0.042	Constant	1.237	0.251	<0.001
Area	2.315	0.644	0.001	Area	2.500	0.346	<0.001	Area	0.880	0.265	0.003
Elevation	−15.431	2.277	<.001					Discharge	0.408	0.146	0.010

Autoregressive models											
Total species richness				Stygobiotic species				Cold stenotherm species			
Variable	Coefficient	Standard error	*p*(t)	Variable	Coefficient	Standard error	*p*(t)	Variable	Coefficient	Standard error	*p*(t)
d				e				f			
Constant	51.602	14.363	0.001	Constant	1.697	1.845	.382	Constant	1.299	0.884	.155
Area	1.444	0.835	0.096	Area	2.858	0.789	.006	Area	0.451	0.388	.260
Elevation	−14.736	4.853	0.005					Discharge	0.337	0.292	.260

OLS regression statistics: a) *n *=* *30, *R* ^2^ = .859; *F *=* *82.282, *p *<* *.001, AICc = 144.128; b) *n *=* *12, *R* ^2 ^= .839; *F *=* *52.242, *p *<* *.001, AICc = 48.844; c) *n *=* *27, *R* ^2^ = .762; *F *=* *38.368, *p *<* *.001, AICc = 74.552. SR regression statistics: d) *n *=* *30, *R* ^2^ (pseudo) = .836 (AICc = 152.630), *R* ^2^ (predictors + space) = .621 (AICc = 177.751); rho = .987, *F *=* *68.707, *p *<* *.001; e) *n *=* *12, *R* ^2^ (pseudo) = .822 (AICc = 55.824), *R* ^2^ (predictors + space) = .958 (AICc = 38.501); rho = .916, *F *=* *46.217, *p *<* *.001; f) *n *=* *27, *R* ^2^ (pseudo) = .668 (AICc = 87.548), *R* ^2^ (predictors + space) = .551 (AICc = 95.696); rho = .983, *F *=* *24.173, *p *<* *.001.

This relationship between stygobiotic species richness and area followed the Gleason exponential model, (*S *=* *1.394 + 2.50 log *A; R* ^2^ = .839, *p *<* *.001). The Arrhenius model provided a poorer fit (log *S* = .172 + .275 log *A*,* R* ^2^ = .750, *p *<* *.001).

For the cold stenotherm species, the OLS best‐fit model included area and discharge (Prediction #5), but these variables were not significant in the autoregressive model (Table 2). For the nonstygobiotic species, the OLS best‐fit model included only elevation as a significant variable (Prediction #4), which however was not significant in the autoregressive model (Table 3). The OLS best‐fit model for the noncold stenotherm species included area and elevation as significant variables, but only elevation was significant in the autoregressive model (Table 3). In general, compared to the independent errors model, explicit consideration of spatial correlations reduced the importance of predictors of overall species richness, cold stenotherm species richness, noncold stenotherm species richness, and nonstygobiotic species richness, but increased the importance of area for the stygobiotic species.

**Table 3:** Parameter values, standard errors and associated probability levels of ordinary least‐squares (OLS, a, b) and spatial autoregressive (SR, c, d) best‐fit models for nonstygobiotic and noncold stenotherm species richness, and log‐transformed environmental variables

OLS models							
Nonstygobiotic species				Noncold stenotherm species			
Variable	Coefficient	Standard error	*p*(t)	Variable	Coefficient	Standard error	*p*(t)
a				b			
Constant	29.306	6.969	<.001	Constant	42.789	5.703	<.001
Area	1.085	0.625	.095	Area	1.396	0.512	.011
Elevation	−0.810	2.211	.001	Elevation	−12.698	1.809	<.001

Autoregressive models							
Nonstygobiotic species				Noncold stenotherm species			
Variable	Coefficient	Standard error	*p*(t)	Variable	Coefficient	Standard error	*p*(t)
c				d			
Constant	28.863	14.097	.051	Constant	43.007	11.438	<.001
Area	−0.003	0.819	.997	Area	0.886	0.665	.194
Elevation	−7.762	4.763	.115	Elevation	−12.252	3.865	.004

OLS regression statistics: a) *n *=* *30, *R* ^2^ = .624; *F *=* *22.443, *p *<* *.001, AICc = 142.361; b) *n *=* *30, *R* ^2^ = .846; *F *=* *74.085, *p *<* *.001, AICc = 130.328. SR regression statistics: c) *n *=* *30, *R* ^2^ (pseudo) = .540 (AICc = 152.363), *R* ^2^ (predictors + space) = .379 (AICc = 161.376); rho = .987, *F *=* *15.844, *p *<* *.001; d) *n *=* *30, *R* ^2^ (pseudo) = .832 (AICc = 136.895), *R* ^2^ (predictors + space) = .689 (AICc = 155.261); rho = .987, *F *=* *63.680, *p *<* *.001.

**Figure 4:**
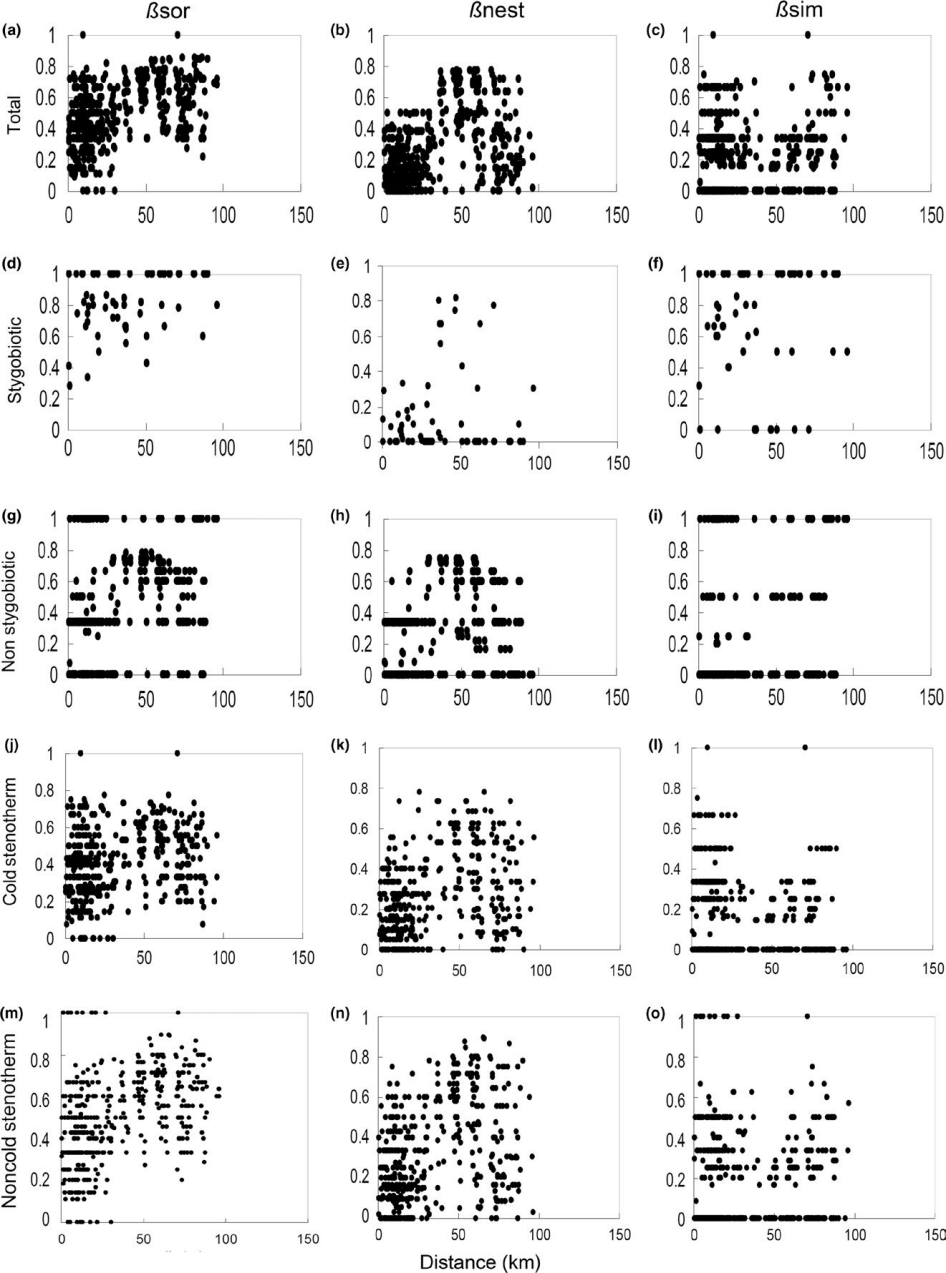
Relations between interspring distance and overall similarity in species composition (ßsor, a, d, g, j, m) and its nestedness (ßnest, b, e, h, k, n) and turnover (ßsim, c, f, i, l, o) components for total species richness (a–c) and stygobiotic (d–f), nonstygobiotic (g–i), cold stenotherm (j–l), and noncold stenotherm (m–o) species, separately

Using the entire data set, both overall species dissimilarity among springs and the nestedness component were correlated with interspring geographical distances (r* *=* *.481, *p *<* *.0001 for ßsor and r* *=* *.437, *p *<* *.0001 for ßnest, respectively), whereas the pure turnover component (ßsim) was not correlated with geographical distances (r* *=* *−.021, *p *=* *.819) (Figure 4a–c). An analysis restricted to cold stenotherm species (Figure 4j‐l) recovered the same patterns: interspring distances were correlated with both overall species dissimilarity and the nestedness component (r* *=* *.384, *p *<* *.001 for ßsor and r* *=* *.233, *p *=* *.011 for ßnest, respectively), but not with the pure turnover (ßsim) component (r* *=* *.194, *p *=* *.064). In the case of the stygobiotic species (Figure 4d–f), we found a marginally significant correlation between interspring geographical distances and overall species dissimilarity (ßsor, r* *=* *.298, *p *=* *.046), whereas no correlation was found for the pure turnover component or the nestedness component (r* *=* *.123, *p *=* *.204 for ßsim and r* *=* *.047, *p *=* *.367 for ßnest, respectively). For the nonstygobiotic species (Figure 4g–i), we found that interspring distances were significantly correlated with overall species dissimilarity (ßsor, r* *=* *.234, *p *<* *.010), pure turnover (ßsim, but negatively, r* *=* *−.182, *p *=* *.032), and nestedness (ßnest, r* *=* *.380, *p *=* *.002). In the case of the noncold stenotherm species (Figure 4m–o), interspring distances were correlated with both overall species dissimilarity and the nestedness component (r* *=* *.441, *p *<* *.001 for ßsor and r* *=* *.381, *p *<* *.017 for ßnest, respectively), but not with the pure turnover (ßsim) component (r* *=* *−.001, *p *=* *.993). Overall, these results support Predictions #3 and #4.

All nestedness indices indicated a significant nestedness for the complete data set and for the cold stenotherm species, which showed a very similar nestedness degree (Table 4). Similarly, both nonstygobiotic and noncold stenotherm species showed significant nestedness values (Table 4). By contrast, all indices but matrix temperature showed a nonnested pattern for the stygobiotic species (Table 4), in accordance with Prediction #3.

**Table 4:** Values of nestedness indices for the matrix including all species and for matrices including only stygobiotic, cold stenotherm, nonstygobiotic and noncold stenotherm species

	Index value	*Z*‐score	Relative nestedness	*p*‐level
All species				
NODF	47.456	21.310	1.026	<.001
T	12.256	−11.594	−0.627	<.001
BR	62.000	−21.356	−0.590	<.001
Spectral radius	11.22	27.134	0.374	<.001
Stygobiotic species				
NODF	25.133	−0.053	−0.006	>.05
T	20.828	−3.402	−0.336	<.001
BR	27.000	−1.495	−0.129	>.05
Spectral radius	4.574	1.241	0.045	>.05
Cold stenotherm species				
NODF	47.031	16.885	1.606	<.001
T	15.374	−4.999	−0.570	<.001
BR	13.000	−10.185	−0.660	<.001
Spectral radius	5.801	10.144	0.365	<.001
Nonstygobiotic species				
NODF	70.096	29.973	1.276	<.001
T	15.105	−9.186	−0.679	<.001
BR	33.000	−17.547	−0.674	<.001
Spectral radius	10.738	25.373	0.327	<.001
Noncold stenotherm species				
NODF	54.545	27.168	1.344	<.001
T	9.991	−9.173	−0.673	<.001
BR	48.000	−15.532	−0.576	<.001
Spectral radius	9.886	20.530	0.376	<.001

## 4 Discussion

In general, we found support for ETIB predictions about overall species richness, with both area and elevation being important predictors. Area was included in all best‐fit models, although its importance varied according to the species ecological categories that were modeled, and was generally diminished when the spatial component was considered. However, for stygobites, area was the only variable retained in the best‐fit models and its importance further increased when the spatial component was included in the analysis (more than 80% of variance in the OLS model and more than 95% of variance in the autoregressive model [predictors + space]). Thus, our first prediction that stygobiotic richness should be influenced by area, but not necessarily by other variables, was largely confirmed. One of the explanations for the species–area relationship is the so‐called habitat diversity hypothesis (Hart & Horwitz, 1991; Kohn & Walsh, 1994). According to this hypothesis, larger areas host more species because they have a greater variety of biotopes, larger environmental gradients and more diverse and abundant resources, etc. (Hortal, Triantis, Meiri, Thébault, & Sfenthourakis, 2009; Hortal et al., 2013; Fattorini et al., 2015). Most of the springs considered in this study are basal karstic springs, where groundwater upwells through large and small fractures of the carbonate bedrock and in the alluvial sediments overlying the aquifer, thus offering several microhabitats for species survival beneath the springbed (Fiasca et al., 2014; Stoch et al., 2016). However, the so‐called passive sampling hypothesis (Connor & McCoy, 1979) can also be evoked. According to this hypothesis, larger islands have more species simply because they “sample” more individuals, and hence more species, from the total pool of immigrants (Connor & McCoy, 1979; Ricklefs & Lovette, 1999; Fattorini et al., 2015). These hypotheses are not mutually exclusive and can be evoked from both an ETIB and a relict perspective. However, in the case of stygobites, the lack of spatial autocorrelation and relationships with other variables suggests that these animals form relict communities (Prediction #2), for which the species–area relationship is not determined by current immigration/extinction processes. Namely, we hypothesize the presence of a “filter effect” exerted by alluvial deposits on species with low dispersal capabilities and that determined a more selective immigration, compared to that of nonstygobites, which have higher dispersal capabilities. This “filter effect” on species washed out by groundwater into the alluvial deposits of the springs led stygobites to be trapped in the sediment matrix (see Galassi et al., 2014). Under this scenario, springs with larger areas may have sampled and retained more species during the “washing” process, whereas rheocrene springs with small areas, where the groundwater rapidly flows downstream, have a much lower “retention potential” for stygobites, resulting in a negligible “filter effect”.

Interestingly, we found that our data were best fit by the Gleason exponential model of the species–area relationship, instead of the Arrhenius power function. It has been hypothesized that the exponential model is more suited when accumulation of new species is relatively slow within an area due to the probability of strong similarity of environmental conditions and species composition of neighboring areas (He & Legendre, 1996). As our system is composed of springs with similar environmental characteristics and species composition, with a relatively small variation in species richness (3–23 species), our results seem to support this hypothesis.

Elevation exerted a negative influence on the total species richness, on nonstygobiotic species richness and on noncold stenotherm species richness. This suggests that nonstygobiotic species and noncold stenotherm species richness values are both higher in lower‐altitude springs, where these generalist species may reach the spring habitats not only from the “mainland” represented by the aquifer but also, and likely predominantly, by means of stepping‐stone dispersal through the surface hydrographic network (Pringle, 2003), in accordance with our Prediction #4. Moreover the intrinsic mosaic feature of the large basal springs offers higher niche availability, thus supporting the co‐occurrence of all the ecological categories analyzed in the present study. Cold stenotherm species richness was positively influenced by area and discharge, thus suggesting that immigration rates of species associated with cold water is enhanced by higher volumes of water, as expected according to Prediction #5. Higher groundwater discharge guarantees cold and stable thermal regime and may favor the development of aquatic vegetation that represents a trophic and spatial microhabitat where some cold stenotherm species live, as observed in Alpine ponds (Ilg & Oertli, 2014). Moreover, most high‐altitude springs are rheocrenic habitats, where the groundwater that feeds the springs flushes out and permanence of water bodies is mainly determined by the *springscape* (Reiss & Chifflard, 2015) described by slope, current velocity, and groundwater discharge (von Fumetti, Nagel, Scheifhacken, & Baltes, 2006; Van Der Kamp, 1995).

Comparisons between OLS regressions and spatial autoregressive models are highly informative. We observed that, with the notable exception of area for stygobites, the use of spatial autoregressive models reduces the importance of predictors selected by OLS regressions. Of course, many environmental parameters are geographically structured, and removing the spatial component should indirectly lead to reduce their influence. Thus, the fact that elevation remained an important predictor of total species richness even in the autoregressive models indicates that this variable exerts an influence that cannot be subsumed by variations in other possible factors associated with spatial position. By contrast, area remained important for stygobites, but not for cold stenotherm and noncold stenotherm species. This suggests that the influence of area on these categories may be due to the influence of variables associated with the positional effect, such as the hydrological unit that can serve as a source pool, the effect of different histories that different parts of the study area may have experienced, or the presence of additional spatially structured environmental conditions that were not considered in this study.

In general, interspring geographical distances influenced both the overall species dissimilarity and the nestedness component, but not the pure turnover component (Prediction #3). This suggests that interspring proximity influences interspring species similarity mainly via variation in species richness; when the effect of species richness is removed, that is, when the pure spatial turnover is considered, the effect of geographical distance disappears. Thus, low turnover and high nestedness values, also returned by NODF and other metrics, are consistent with an equilibrium scenario mainly regulated by immigration and extinction. However, in the case of stygobiotic species, as originally predicted, we found only a marginally significant correlation between interspring geographical distances and overall species dissimilarity, whereas no correlation was found for the pure turnover component or the nestedness component. Moreover, the species per spring matrix of stygobiotic species was not significantly nested for all used metrics, except for T, which is however known to be prone to type I error (Ulrich et al., 2009). The lack of nestedness results from unexplained species presences or absences and thus indicates that the distribution of stygobiotic species is largely idiosyncratic and was influenced by biogeographic events different from the immigration/extinction processes affecting the other species. For example, geological and past hydrological processes may have led to alternate phases of isolation and connection of the aquifers (Bauzà‐Ribot, Jaume, Fornós, Juan, & Pons, 2011), which may explain the co‐occurrence or disjunct distribution of stygobiotic species in aquifers that are now isolated. Distributional and evolutionary relicts are common among stygobites inhabiting subsurface spring environments, where these species can found conditions (such as a constant thermal regime, an oligotrophic status, and permanent darkness) that mirror those typical of their original habitat, represented by the aquifer feeding the springs where they are trapped (Galassi et al., 2009; Holsinger, 1988; Humphreys, 2000).

## 5 Conclusions

Springs are known to serve as ecological refugia (variable in time and space) for surface‐water and groundwater invertebrates (Cantonati et al., 2012), and also as evolutionary refugia (Botosaneanu, 1995; Davis et al., 2013) especially for stygobites whose primary habitat is represented by the aquifer itself hosting narrow endemic and paleoendemic taxa, the latter with disjunct distribution across springs fed by different aquifers. Whereas nonstygobiotic species show distributional patterns mainly influenced by real‐time ecological constraints and which are consistent with ETIB predictions, stygobiotic species show spot distributions among spring habitats, being the only survivors of ancient lineages that become extinct in surface‐water bodies, or, alternatively, as a result of fragmentation of a wider original distribution. In both cases, springs represented for stygobites conservative environments that “trapped” species originally depending on the stable groundwater environments of the aquifers below the spring cup (Galassi et al., 2009; Gibert & Deharveng, 2002).

## Conflict of Interest

None declared.
